# Experiments and Preliminary Modeling of Chloride Ingress in Concrete Interfaces Under Marine Drying–Wetting Environment

**DOI:** 10.3390/ma19143123

**Published:** 2026-07-21

**Authors:** Yuanyuan Cheng, Jinlong Zhang, Zhiyuan Zhao, Peng Ni, Qingxin Meng, Jinjun Guo, Hongrui Chen, Yazhou Jiang, Kun Wang

**Affiliations:** 1 School of Water Conservancy and Transportation, Zhengzhou University, Zhengzhou 450001, China; chengyuanyuan@gs.zzu.edu.cn (Y.C.); zjla7788@163.com (J.Z.); m17637216871@163.com (Z.Z.); chenhr1031@gmail.com (H.C.); 19513301780@163.com (Y.J.); 2 China State Construction Engineering Corporation Seventh Division Co., Ltd., Zhengzhou 450004, China; 3 State Key Laboratory of Tunnel Boring Machine and Intelligent Operation, Zhengzhou 450001, China

**Keywords:** bond interface, new-to-old concrete, chloride penetration, drying–wetting cycles, bond interface influence coefficient, chloride diffusion coefficient

## Abstract

A bond interface between new and old concrete is inherently present in bridge widening and rehabilitation projects. Under marine environmental conditions, this interface provides a preferential pathway for chloride ion transport, thereby accelerating chloride-induced corrosion of the bridge structure. Although the mechanical bonding performance of such interfaces has been extensively investigated, the durability of new-to-old concrete systems remains significantly inferior to that of monolithic concrete, particularly in terms of resistance to chloride penetration. In this study, chloride erosion tests on new-to-old concrete specimens were conducted under drying–wetting cycle conditions to investigate the influence of the bond interface on the spatial distribution and temporal evolution of chloride concentration. The results indicate that the chloride concentration at the bond interface is significantly higher than that in other regions. This leads to a dual transport mechanism, where chloride ions not only diffuse inward perpendicular to the exposed surface but also migrate laterally from the interface into the adjacent concrete driven by concentration gradients. Based on these findings, an interface influence coefficient is proposed to quantify the effect of the bond interface on chloride transport capacity. This coefficient exhibits a strong fit with the GaussAmp function. Furthermore, a diffusion coefficient model for new-to-old concrete incorporating the effect of the bond interface is established.

## 1. Introduction

The rapid increase in the number of motor vehicles, together with the expansion of travel demand, has imposed increasingly stringent requirements on roadway traffic capacity [1,2,3]. As a critical component of highway transportation infrastructure, bridges significantly influence traffic flow and transport efficiency [4]. However, the majority of bridges constructed during the last century were designed to relatively low load standards, rendering them inadequate to cope with the current surge in traffic demand. Consequently, there is an urgent need for their rehabilitation and upgrading. Among the available solutions, bridge widening offers a highly economical and feasible approach to enhance bridge traffic capacity and service level without interrupting the operation of the existing bridge [5].

The widening of an existing bridge inevitably creates a composite structure comprising new and old concrete joined by a bond interface. The microstructural heterogeneity of this bond interface gives rise to disparities in both physical and chemical properties between the two concrete components. Consequently, the bond strength between new and old concrete is compromised, resulting in lower overall mechanical performance compared to monolithic cast-in-place concrete. Chloride ingress into concrete occurs through multiple mechanisms, including diffusion, permeation, and capillary absorption in marine environments. This aggressive attack is further accelerated in regions characterized by strong tidal action, where sea breezes induce chloride accumulation on the concrete surface and tidal cycles generate drying–wetting alternations [6,7,8]. Therefore, durability issues in new-to-old concrete associated with chloride penetration become particularly critical [9,10,11]. Previous studies have shown that the bond interface between new and old concrete contains numerous microcracks and voids along with incomplete hydration, resulting in a lower degree of compactness and a more porous structure compared to the bulk concrete matrix [12]. Consequently, the bond interface is generally regarded as a weak region within the structure [13,14,15,16,17]. It significantly influences chloride transport and must be considered a key factor in the durability assessment of bridge widening structures.

In recent years, several studies have investigated chloride-induced deterioration in new-to-old systems. Udaipurwala et al. [18] reported that the interfacial bond strength is insufficient to resist chloride ingress. Li et al. [19] examined the effects of joint types and stress levels on the chloride resistance of jointed concrete, demonstrating that monolithic concrete exhibits better resistance than concrete with various joint configurations. Li and Luo [20] explored chloride transport behavior at the bond interface between precast and cast-in-place concrete with varying roughness levels through long-term immersion tests. The results showed that chloride concentrations in the interfacial zone are significantly higher than those in adjacent regions. The lowest permeability was observed in specimens with moderate surface roughness, whereas both lower and higher roughness levels resulted in increased chloride penetration. Huang et al. [21] investigated the influence of the new-to-old concrete bond interface on the corrosion of reinforcing steel exposed to chloride attack using an electrochemical accelerated corrosion method, considering both natural immersion and drying–wetting cycle conditions. The results indicated that steel corrosion in concrete occurs predominantly at the bonded interface, while corrosion in other regions is relatively uniform and less severe, particularly under drying–wetting cycles. Zhang et al. [22] utilized ultrasonic nondestructive testing (UNDT) and the RCM method to investigate the bond performance and chloride penetration resistance at the new-to-old concrete interface. The findings showed that when the compressive strength of the new concrete exceeds that of the old concrete, the properties of the old concrete primarily govern chloride ingress at the interface. Collectively, these studies have provided a fundamental understanding of the transport behavior at the bond interface of new-to-old concrete. However, most of these investigations have focused on the analysis of experimental phenomena, lacking a systematic theoretical framework for quantification.

To predict the durability of concrete during its service life, chloride diffusion models based on Fick’s second law have been widely adopted. These models have gradually incorporated factors such as time-dependent diffusion coefficients, temperature and humidity variations, and the effects of mineral admixtures to more accurately describe chloride transport behavior under complex environmental conditions [23,24,25]. Tang et al. [26,27] proposed a time-dependent diffusion model that describes the gradual decrease in the chloride diffusion coefficient of concrete with increasing age through an aging factor. Andrade et al. [28,29] developed a practical fitting method for chloride concentration profiles exhibiting internal maxima and analyzed the influence of the concrete “skin effect” on chloride diffusion behavior based on a two-layer diffusion model. Van der Zanden et al. [30] developed a chloride transport model for concrete subjected to annual drying–wetting cycles that accounts for both convective and diffusive effects, enabling effective prediction of the chloride concentration distribution within concrete. However, most existing models are predicated on the assumption of material homogeneity, with limited consideration given to the distinctive transport behavior occurring in locally heterogeneous regions such as bond interfaces. While previous studies on new-to-old concrete have characterized the influence of the bond interface on chloride transport, a theoretical framework that quantifies this influence and integrates it into a comprehensive diffusion model remains lacking. Consequently, accurately representing the actual corrosion process of widened bridges under service environments remains a challenge.

This study aims to thoroughly elucidate the chloride transport characteristics at the bond interface between new and old concrete. By subjecting the new and old concrete to drying–wetting cycle conditions to accelerate the chloride permeation, the spatial distribution and temporal evolution characteristics and laws of chloride ions at the bond interface were extracted and analyzed using a potentiometric titration method. Subsequently, based on a modified form of Fick’s second law, a method for calculating the influence coefficient of the new-to-old concrete bond interface is proposed to quantitatively characterize the effect of the interface on chloride transport. Furthermore, a chloride diffusion coefficient model for new-to-old concrete is established that explicitly incorporates the influence of the bond interface. This research seeks to provide a clearer understanding of the chloride transport process at the new-to-old concrete interface under drying–wetting cycles and to serve as a reference for future investigations in related fields.

## 2. Materials and Methods

### 2.1. Raw Materials

In this study, P.O 42.5 Portland cement, Class F Grade I fly ash, and S95 ground granulated blast-furnace slag (GGBS) were used. These materials were supplied by Yueqing Hailuo Cements Co., Ltd. (Wenzhou, China), Yueqing Jialong Fly Ash Co., Ltd. (Wenzhou, China), and Lubi New Materials Co., Ltd. (Rizhao, China), respectively. Their detailed properties are presented in Table 1, Table 2, Table 3 and Table 4. Natural river sand was employed as the fine aggregate, with a fineness modulus of 2.7 and an apparent density of 2630 kg/m^3^. Natural crushed stone with a particle size ranging from 5 to 25 mm and an apparent density of 2680 kg/m^3^ was used as the coarse aggregate. A KC-NF-1 polycarboxylate superplasticizer (water-reducing rate of 23.4%) from Wenzhou Kaike Building Materials Co., Ltd. (Wenzhou, China) was adopted. Tap water was used for mixing throughout the experiments.

### 2.2. Specimen Preparation

**Figure 1:**
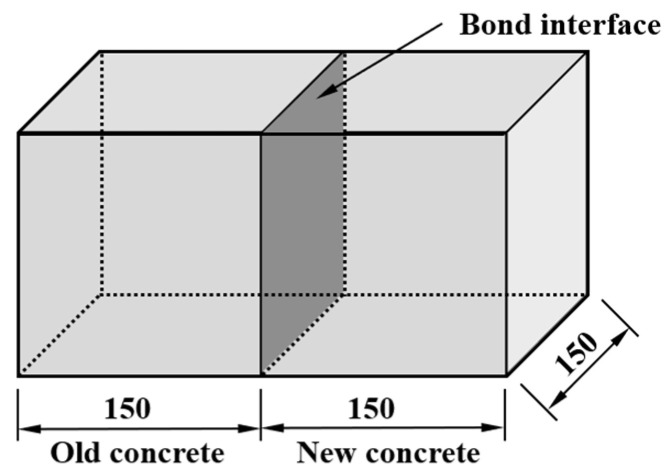
New-to-old concrete specimen.

In this study, composite new-to-old concrete specimens were fabricated by combining two identical cubic blocks with a total assembly size of 150 mm × 150 mm × 300 mm, as illustrated in Figure 1. The mix proportions were determined based on an actual bridge widening project. A water-to-binder ratio of 0.37 was used for the old concrete, whereas 0.39 was adopted for the new concrete. The detailed mix designs are presented in Table 5. The old concrete was cast six months in advance of the new concrete to allow sufficient hydration and to obtain a stable material state. This experimental design aims to simulate a new-to-old concrete composite under marine drying–wetting conditions, with particular emphasis on the role of the bond interface in chloride ingress behavior.

**Figure 2:**
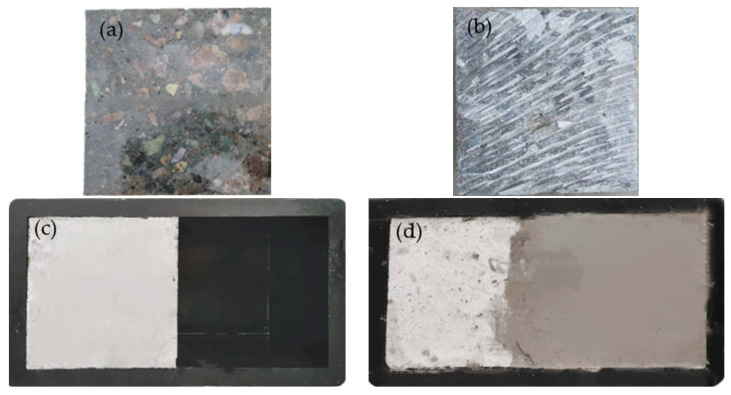
Specimen preparation procedure: (**a**) cutting of the old concrete, (**b**) roughening of the old concrete cut surface, (**c**) placement of the old concrete into the mold, and (**d**) casting of the new concrete.

The new and old concrete specimens were prepared in accordance with the standard for mix proportion design of ordinary concrete (JGJ55–2011) [32]. The casting process and interface treatment are illustrated in Figure 2. First, old concrete specimens with dimensions of 150 mm × 150 mm × 300 mm were cast. After 6 months of standard curing, each specimen was cut transversely at mid-height using a cutting machine, resulting in two 150 mm × 150 mm × 150 mm half-specimens. The cut surface of the old concrete was treated using an angle grinder. The surface roughness after treatment was measured by the sand-filling method, yielding an average depth of 5.1 mm, which satisfied the bond quality requirements. After cleaning the surface and removing loose debris, the old concrete block was set in the mold with the bonding face centered and vertical. Subsequently, the new concrete was cast against the prepared bond interface. The surfaces of both the new and old concrete were covered with plastic film for 24 h. The specimens were then demolded and cured for 28 days in a standard environment at 20 ± 2 °C with a relative humidity of 95%.

### 2.3. Chloride Erosion Test

To simulate the service environment of widened bridge piers in practical engineering, a drying–wetting cycle method was adopted for the chloride erosion test. Numerous studies have investigated the influence of drying–wetting cycles on the transport behavior of aggressive ions in concrete [33,34,35,36]. Considering the effect of an aggressive drying–wetting environment, a drying-to-wetting time ratio of 5:1 was selected, with each complete cycle lasting 24 h. The specimens were subjected to 30, 60, and 90 drying–wetting cycles, corresponding to exposure durations of 30, 60, and 90 days, respectively. Three parallel specimens were prepared for each exposure duration. According to the modified Fick’s second law, increasing the salt concentration shortens the required experimental duration. A 10% NaCl solution was adopted as the erosion solution to accelerate the chloride erosion rate. The sodium chloride was supplied by Shandong Enlin Biotechnology Co., Ltd. (Qingdao, China).

To ensure that chloride ions penetrated into the concrete along the bond interface of new and old concrete in a one-dimensional transport manner, only the casting bottom surface of the specimen was exposed as the erosion surface, while the remaining five surfaces were fully sealed by applying an epoxy resin coating. Subsequently, the specimens were immersed in a 10% NaCl solution at 20 ± 2 °C for 4 h, then removed and allowed to dry naturally for 20 h. This procedure was repeated cyclically. The chloride solution was renewed every seven drying–wetting cycles to maintain a constant concentration.

After the specified exposure duration was reached, the concrete specimens were air-dried under ambient conditions for 24 h. Powder samples were collected by drilling using an intermittent procedure. To avoid the influence of boundary effects, powder sampling by drilling was performed from the interior of the specimen toward the exterior. Prior to sampling, each specimen was cut perpendicular to the exposed surface using a water-cooled cutting machine, yielding two rectangular concrete blocks with dimensions of 150 mm × 75 mm × 300 mm.

**Figure 3:**
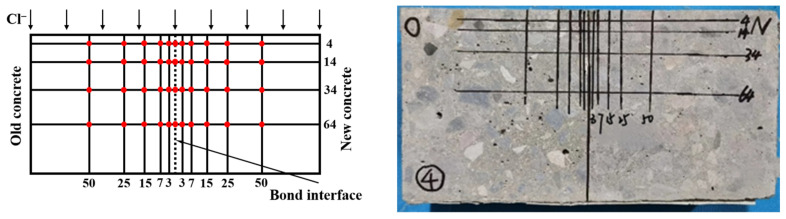
Sampling point arrangement (mm) and direction of chloride ion ingress from the exposed surface.

Sampling points were designated in the old concrete, the bond interface, and the new concrete, with a denser arrangement of sampling points near the bond interface. In the horizontal direction (perpendicular to the bond interface), the distances of the sampling points from the bond interface were 0 mm, 3 mm, 7 mm, 15 mm, 25 mm, and 50 mm. Owing to the drying–wetting cycle action, a convection zone develops in the near-surface layer of the concrete subjected to chloride erosion. The distance from the concrete surface to the point of maximum chloride content is generally defined as the convection zone depth, denoted as Δy. The value of Δy is influenced by factors such as environmental conditions, material properties, and exposure time, and no universally accepted standard exists for its determination. Based on previous studies [37,38,39], a convection zone depth of y=4 mm was used in this work. Accordingly, sampling points in the vertical direction (parallel to the bond interface) were arranged at distances of 4 mm, 14 mm, 34 mm, and 64 mm from the erosion surface. Figure 3 illustrates the sampling point layout.

**Figure 4:**
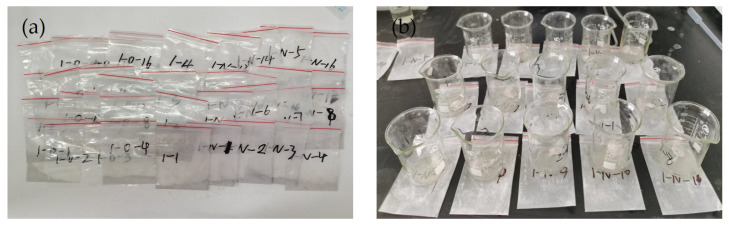

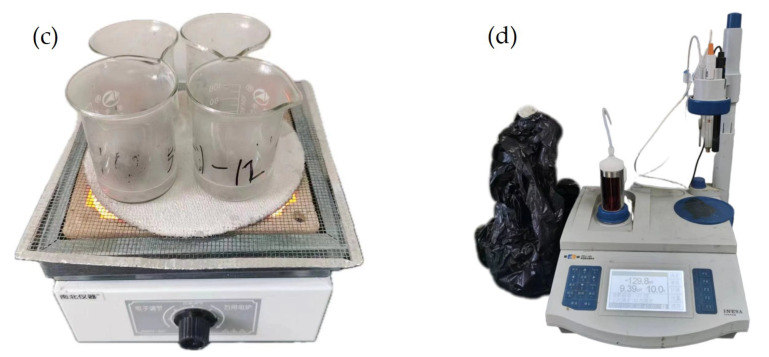
Procedure for chloride content determination using potentiometric titration: (**a**) preparation of concrete powder samples, (**b**) drying of the powder samples, (**c**) heating of the sample solution, and (**d**) determination of the chloride content.

The chloride concentration in the eroded concrete was determined using a ZDCL-2 chloride ion automatic potentiometric titrator (Shanghai INESA Scientific Instrument Co., Ltd., Shanghai, China), following the chloride ion testing methods specified in the Chinese standards JGJ/T 322-2013, GB/T 50476-2019, and GB/T 50344-2019 [40,41,42]. First, the collected concrete powder was dried in an oven at 60 °C for 24 h. Subsequently, 1 g of the powder was dissolved in 20 mL of distilled water, heated to a boil for 1–2 min, and then cooled to room temperature. After that, phenolphthalein and dilute sulfuric acid were added to the solution, followed by dilution with distilled water to 60 mL, and a magnetic stir bar was placed into the solution. Finally, the beaker was placed on the titration platform of the automatic potentiometric titrator. A chloride ion electrode and A calomel electrode were immersed in the sample, the stirrer was turned on, and the solution was titrated gradually with a 0.05 mol/L silver nitrate standard titration solution until the endpoint was reached, as illustrated in Figure 4. All chemical reagents used in the tests were of analytical grade and supplied by Sinopharm Chemical Reagent Co., Ltd. (Shanghai, China).

The mass fraction of chloride ions in the concrete specimen was calculated according to Equation (1).

(1) $$P=\frac{C_{{\mathrm{AgNO}}_{3}}\times V\times 35.45}{m\times 1000}\times 100\%$$

where *P* is the mass fraction of chloride ions; $C_{{\mathrm{AgNO}}_{3}}$ is the concentration of the silver nitrate standard titration solution, which is 0.05 mol/L in this study; *V* is the volume of the silver nitrate standard solution consumed during titration; and *m* is the mass of the powder sample taken.

## 3. Results and Discussion

### 3.1. Distribution Characteristics of Chloride Ions in the Horizontal Direction

**Figure 5:**
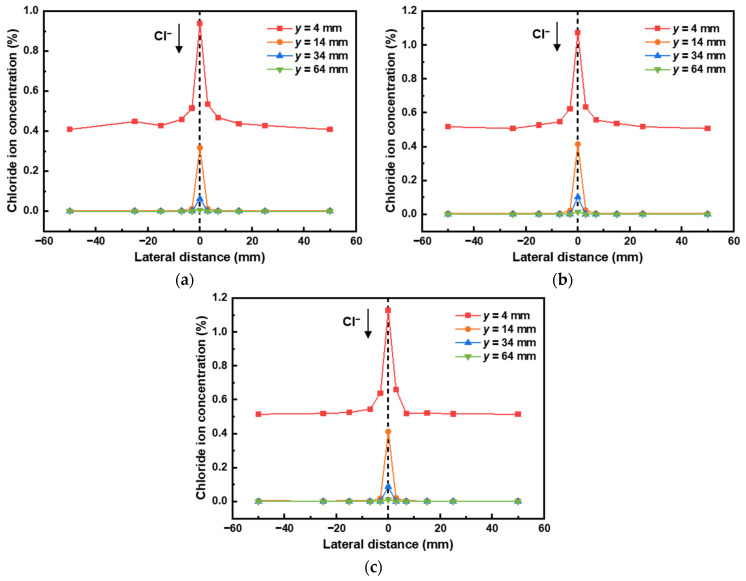
Horizontal distribution of chloride concentration under different erosion durations: (**a**) 30 d, (**b**) 60 d, and (**c**) 90 d. Negative values on the abscissa represent the old concrete region, positive values represent the new concrete region, and s=0 denotes the bond interface between the new and old concrete (dashed line in all figures). The arrows indicate the direction of chloride ion ingress from the exposed surface.

Figure 5 presents the variation in chloride concentration with distance from the bond interface (denoted as s). The peak chloride concentration occurs at the bond interface between the new and old concrete. As the distance from the bond interface increases, the chloride concentration decreases and then gradually stabilizes. The presence of the bond interface results in significantly higher chloride concentrations within 15 mm of the interface than in other regions.

Owing to the hydrophilicity of the old concrete, a locally higher water-to-cement ratio develops in the new concrete adjacent to the bond interface. Furthermore, the aggregate–cement paste interface at the bond interface—comprising aggregates, cement stone, and the new-to-old concrete junction—differs from that in monolithic concrete. Aggregates accumulate on the surface of the old concrete, preventing the cement paste from fully penetrating the voids, a phenomenon known as the “paste deficiency” effect. These factors have been suggested in previous studies [43,44] to result in increased porosity and reduced density at the bond interface, making it easier for chloride ions to ingress through this region.

**Figure 6:**
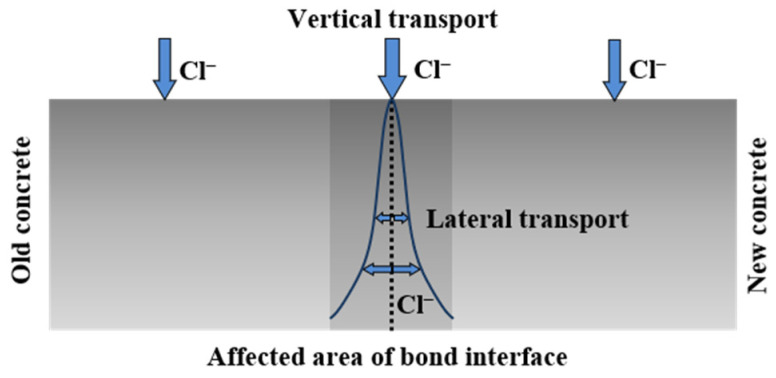
Schematic diagram of chloride transport in new and old concrete.

In the bond interface zone, the chloride transport rate is relatively high, generating a concentration gradient between the interface and the adjacent concrete on both sides. Driven by this concentration gradient, chloride ions laterally diffuse from the bond interface into the surrounding concrete (see Figure 6), forming a two-dimensional transport pattern. Consequently, the chloride concentration exhibits an inverted “V”-shaped distribution near the bond interface. As the distance from the bond interface increases, the concrete structure gradually becomes more homogeneous, the influence of the bond interface diminishes, and the variation in chloride concentration progressively decreases.

**Figure 7:**
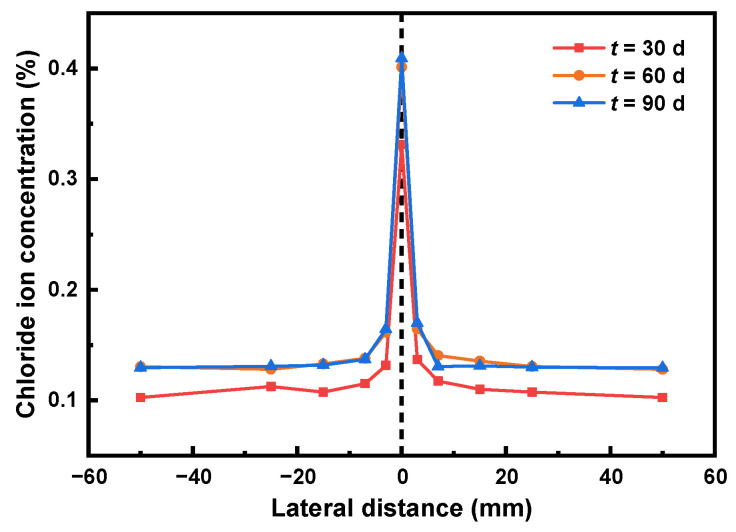
Horizontal distribution of depth-averaged chloride concentration under different erosion durations.

Figure 7 illustrates the influence of erosion duration on chloride transport, based on the average chloride concentration analyzed at different depths for each specimen. As the erosion duration increases, the chloride concentration at the same location in both new and old concrete increases, albeit with a gradually decelerating trend. Chlorides are transported in concrete primarily by diffusion, migrating from regions of high concentration to regions of low concentration. The greater the concentration gradient, the faster the diffusion rate. At the early stage of erosion, the concentration gradient between the concrete surface layer and the interior is substantial, resulting in a relatively rapid chloride transport rate. As the erosion duration increases, the chloride concentration gradient gradually diminishes, leading to a decrease in the transport rate.

### 3.2. Distribution Characteristics of Chloride Ions in the Vertical Direction

**Figure 8:**
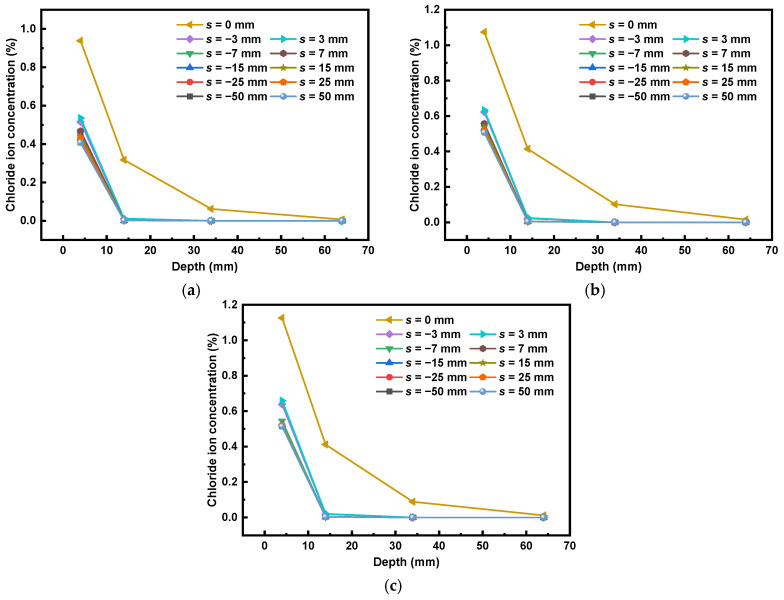
Vertical distribution of chloride concentration under different erosion durations: (**a**) 30 d, (**b**) 60d, and (**c**) 90d.

Figure 8 presents the variation in chloride concentration with erosion depth (denoted as y) in both new and old concrete. Under different erosion durations, the chloride concentration decreases with increasing erosion depth. At the same distance from the bond interface, the amplitude of chloride concentration variation gradually diminishes as the erosion depth increases.

**Figure 9:**
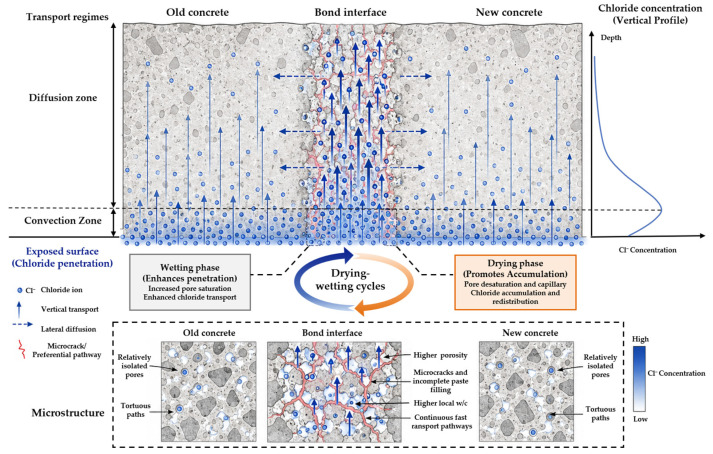
Schematic illustration of chloride transport at the new-to-old concrete bond interface under drying–wetting cycles, where the interface width is exaggerated for clarity.

Under drying–wetting cycle conditions, chloride transport in the shallow layer of concrete is governed by the coupled effects of capillary absorption and diffusion. As the depth increases, the capillary absorption effect gradually weakens, and chlorides migrate deeper, primarily driven by the concentration gradient, resulting in a progressively decreasing transport rate. The bond interface effect remains evident across different depth ranges, indicating that its influence is not limited to the shallow layer of concrete. This underlying transport mechanism is schematically illustrated in Figure 9. The accelerated wetting–drying exposure conditions adopted in this study should be taken into consideration when interpreting the present results in relation to long-term field chloride transport behavior.

### 3.3. Influence Coefficient of the New-to-Old Concrete Bond Interface

#### 3.3.1. Calculation Model for the Bond Interface Influence Coefficient

Fick’s second law is commonly employed to describe chloride transport behavior in concrete, as presented in Equation (2). In this framework, chlorides are simplified as diffusing into a one-dimensional semi-infinite medium, with the chloride concentration exhibiting a gradient along the specimen length from the concrete surface to the interior. When the diffusion coefficient is constant, the analytical solution to Equation (2) can be expressed as Equation (3), subject to the appropriate initial and boundary conditions (i.e., $C(0,t)=C_{s}$, $C(\infty ,t)=C_{0}$, $C(y,0)=C_{0}$) [45,46]:

(2) $$\frac{\partial C}{\partial t}=\frac{\partial }{\partial y}\left(D\frac{\partial C}{\partial y}\right)$$

(3) $$C\left(y,t\right)=C_{0}+(C_{s}-C_{0})\left[1-erf\left(\frac{y}{2\sqrt{Dt}}\right)\right]$$

where *D* is the apparent chloride diffusion coefficient; $C\left(y,t\right)$ is the chloride concentration at depth y from the concrete surface at time *t*; $C_{s}$ and $C_{0}$ are the surface chloride concentration and the initial chloride concentration, respectively; *t* is the diffusion time; y is the depth from the concrete surface to the testing location; and *erf* is the error function.

The apparent chloride diffusion coefficient is governed by the material properties of the concrete and the exposure environment [47,48]. Within a given structural region, chloride transport is influenced by specific factors, which may be dominated by a single factor or result from the coupled effects of multiple factors. To better simulate the chloride transport process in concrete, the diffusion coefficient is defined as a variable dependent on different influencing factors. Consequently, during chloride transport within the concrete structure, the diffusion coefficient exhibits a multiplicative coupling effect, which can be described as the product of a function of the dominant factor(s) and the diffusion coefficient under initial conditions, expressed as

(4) $$D_{{\mathrm{Cl}}^{-}}=D_{{\mathrm{Cl}}^{-},t}\times f(a_{1})\times f(a_{2})\times \cdots \times f(a_{i})$$

where $D_{{\mathrm{Cl}}^{-}}$ is the chloride diffusion coefficient considering the coupled effects of multiple influencing factors; $D_{{\mathrm{Cl}}^{-},t}$ is the initial chloride diffusion coefficient at time *t*; and $f(a_{i})$ is the *i*-th dominant influencing factor.

As revealed by the analyses in Section 3.1 and Section 3.2, the chloride concentration exhibits significant differences among the bond interface region, the new concrete, and the old concrete. In this study, the external environment and the material properties of the concrete were kept constant; therefore, the bond interface is identified as the primary factor responsible for these differences. To quantify its effect on chloride transport, a bond interface influence coefficient, denoted as f(i), is introduced. Assuming that the chloride diffusion coefficient of ordinary concrete is denoted as $D_{0}$, the effect of the bond interface on chloride transport in the new-to-old concrete can be expressed as

(5) $$f(i)=\frac{D_{i}}{D_{0}}$$

where $D_{i}$ is the chloride diffusion coefficient in the bond interface zone.

#### 3.3.2. Apparent Chloride Diffusion Coefficient

For concrete structures located in splash zones, tidal zones, and similar environments, chloride ingress is a coupled process owing to the drying–wetting cycle action. Within the concrete interior, chloride transport is dominated by diffusion, whereas at the concrete surface, both convection and diffusion effects coexist. As a result, a convection zone develops at the concrete surface, where the peak chloride concentration does not appear at the exposed surface. In other words, chloride penetration in the surface layer does not conform to Fick’s second law. To address this issue, chloride ingress into concrete can be analyzed by considering only the diffusion zone where the concentration profile exhibits a decreasing trend [29,49].

The International Federation for Structural Concrete (fib) [50] proposed a modified Fick’s second law model, as presented in Equation (6). In this model, the surface chloride concentration is replaced by the peak chloride concentration in the convection zone, denoted as $C_{s,\Delta y}$, enabling the prediction of chloride transport in concrete under drying–wetting cycle conditions.

(6) $$C(y,t)=C_{0}+(C_{s,\Delta y}-C_{0})\left[1-erf\left(\frac{y-\Delta y}{2\sqrt{Dt}}\right)\right]$$

Based on the above analysis, at the bond interface (s=0), chloride ions not only undergo one-dimensional transport from the concrete surface along the depth direction but also laterally diffuse from the bond interface into the adjacent concrete on both sides, forming a two-dimensional diffusion pattern. This behavior is described by the following equation:

(7) $$\frac{\partial C}{\partial t}=D\frac{\partial^{2}C}{\partial s^{2}}+D\frac{\partial^{2}C}{\partial y^{2}}$$

The boundary conditions are given as $C(s,y,0)=C_{0}$, C(0,y,t)=C(y,t) and $C(s,0,t)=C_{s,\Delta y}$. The corresponding analytical solution can be expressed as

(8) $$C\left(s,y,t\right)=C_{0}+(C_{s,\Delta y}-C_{0})\left[1-erf\left(\frac{s}{2\sqrt{D_{i}\cdot t}}\right)erf\left(\frac{y-\Delta y}{2\sqrt{D_{i}\cdot t}}\right)\right]$$

where $C\left(s,y,t\right)$ is the chloride concentration at depth y from the concrete surface and distance s from the bond interface at time *t*; $C_{s,\Delta y}$ is the peak chloride concentration in the convection zone (Δy=4 mm); $D_{i}$ is the apparent chloride diffusion coefficient at the bond interface; *t* is the diffusion time; y is the diffusion depth; and s is the distance from the bond interface.

In addition, beyond a certain distance from the bond interface, chloride transport can be reasonably approximated as one-dimensional diffusion along the depth direction. In this region, the diffusion coefficient can be determined using Equation (6).

#### 3.3.3. Analysis of the Bond Interface Influence Coefficient

**Figure 10:**
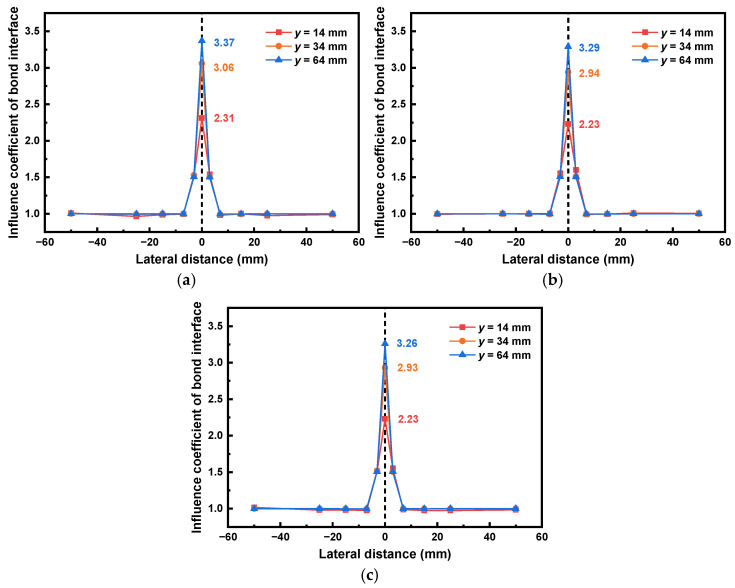
Lateral distribution of the bond interface influence coefficient under different erosion durations: (**a**) 30 d, (**b**) 60 d, and (**c**) 90 d.

As revealed by the analysis in Section 3.1, the influence zone of the bond interface on chloride transport extends within ±15 mm from the interface. Beyond this range, the chloride concentration tends to stabilize at a constant value. Therefore, when the distance from the bond interface exceeds 15 mm, it is considered that chloride transport is essentially unaffected by the bond interface. The chloride diffusion coefficients at locations ±50 mm from the bond interface in the concrete on both sides were averaged to obtain a representative value of the stable chloride diffusion coefficient within the concrete, denoted as $D_{0}$. Figure 10 presents the bond interface influence coefficient f(i) at different locations in the new and old concrete.

At the same erosion depth, the bond interface influence coefficient gradually increases as the distance from the bond interface decreases, reaching its peak exactly at the bond interface. This result indicates that the chloride diffusion coefficient attains its maximum value at the bond interface, signifying the highest chloride transport capacity in this region. Furthermore, in the vicinity of the bond interface, the effectiveness of the bond interface on chloride transport gradually diminishes with increasing distance from the interface. This trend is consistent with the experimental observations described above.

As the erosion depth increases, the bond interface influence coefficient exhibits a gradually increasing trend in the bond interface, the old concrete, and the new concrete, reaching its maximum value when the erosion depth attains 64 mm. This phenomenon can be attributed to the higher porosity in the bond interface zone, which facilitates faster chloride transport in this region. As erosion depth increases, the chloride concentration at the bond interface decreases to a relatively smaller extent. In contrast, in the concrete regions on both sides away from the bond interface, the chloride transport rate along the depth direction is slower, and the chloride concentration decreases to a relatively larger extent as the erosion depth increases. Consequently, the difference in chloride concentration between the bond interface zone and the adjacent concrete on both sides gradually widens with increasing depth, leading to an enhanced lateral transport effect and, in turn, an increase in the bond interface influence coefficient.

**Figure 11:**
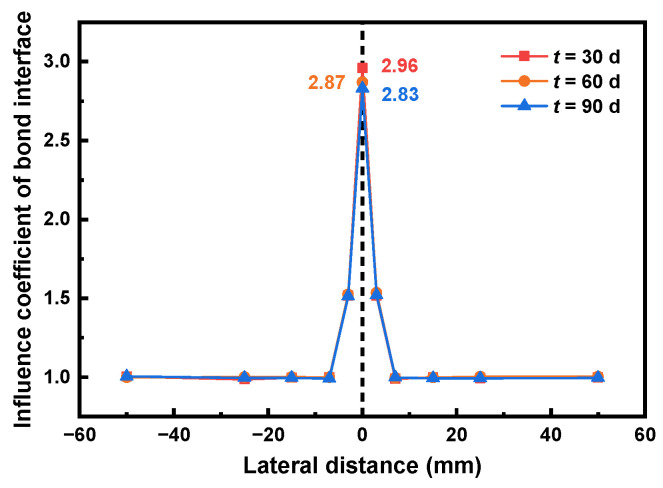
Influence of erosion duration on the distribution of the bond interface influence coefficient at different locations.

Similarly, the bond interface influence coefficients at different depths were averaged for each specimen to elucidate the effect of erosion duration on the bond interface influence coefficient, as shown in Figure 11. As the erosion duration increases, the bond interface influence coefficient at the same location in the bond interface zone exhibits a decreasing trend, with the trend gradually decelerating. This is because chloride ions continuously migrate into the concrete over time. At a given erosion depth, the concentration gradient of chloride ions between the bond interface and the adjacent concrete on both sides progressively increases, leading to an increasingly rapid lateral transport rate of chlorides. This effect counteracts the concentration gradient, causing it to diminish over time, and consequently, the bond interface influence coefficient decreases correspondingly.

## 4. Chloride Diffusion Coefficient Model for New and Old Concrete

### 4.1. Model Establishment

Concrete, composed of cement paste, aggregates, and the interfacial transition zone (ITZ), is a multiphase composite. Consequently, its performance depends on the properties of each constituent phase and their interrelationships. Based on this three-phase composition of concrete, porosity is adopted as a key parameter to establish a chloride diffusion coefficient model for new and old concrete that incorporates the effect of the bond interface.

#### 4.1.1. Chloride Diffusion Coefficient of Cement Mortar

The diffusion performance of chloride ions in cement mortar is related to its porosity. According to Garboczi and Bentz [51], the chloride diffusion coefficient of the cement mortar matrix is related to its porosity as expressed in Equation (9).

(9) $$D_{m}=D_{0}\times \left[0.001+0.07\varphi +1.8H\times {\left(\varphi -\varphi_{th}\right)}^{3}\right]$$

where $D_{m}$ is the chloride diffusion coefficient in the cement mortar matrix; $D_{0}$ is the chloride diffusion coefficient in aqueous solution, taken as 2.032 × 10^−9^ m^2^/s at 25 °C; φ is the porosity of the cement mortar matrix; *H* is the Heaviside function, where *H* = 1 when $\varphi >\varphi_{th}$ and *H* = 0 otherwise; and $\varphi_{th}$ is the critical porosity threshold, taken as 0.18.

The porosity of cement mortar is influenced by the water-to-cement ratio and the degree of hydration. Sun et al. [52] refined the relationship among these variables through steady-state chloride accelerated diffusion tests as follows:

(10) $$\varphi =\frac{w/c-0.17\alpha_{w}}{w/c+0.32}$$

(11) $$\alpha_{w}=0.716t_{c}^{0.0901}\exp\left[-0.103t_{c}^{0.0901}/\left(w/c\right)\right]$$

where w/c is the water-to-cement ratio of the cement mortar; $\alpha_{w}$ is the degree of hydration of the cement mortar; and $t_{c}$ is the curing time, taken as $t_{c}=28\;d$.

#### 4.1.2. Effect of Coarse Aggregate on the Diffusion Coefficient

The tortuosity effect, dilution effect, interfacial transition zone (ITZ) effect, and percolation effect of coarse aggregates in concrete influence chloride diffusion. Among these, the tortuosity and dilution effects reduce chloride diffusion efficiency, whereas the ITZ and percolation effects increase it. Yang and Cho [53] reported that the percolation effect is negligible when the volume fraction of coarse aggregates is less than or equal to 0.5. Therefore, only the tortuosity, dilution, and ITZ effects of coarse aggregates are considered in this study.

(1) Dilution effect. It is generally recognized that chloride ions cannot migrate through coarse aggregates. The incorporation of coarse aggregates into cement-based materials reduces the spatial region available for chloride transport, thereby lowering the chloride diffusion coefficient. Studies have shown that the magnitude of the dilution effect is related to the volume fraction of coarse aggregates: the larger the volume fraction of coarse aggregates, the greater the dilution effect. When the dilution effect of coarse aggregates is taken into account, the chloride diffusion coefficient in concrete is given by

(12) $$D_{D}=D_{m}\left(1-V_{f}\right)$$

where $D_{D}$ is the chloride diffusion coefficient in concrete considering only the dilution effect, and $V_{f}$ is the volume fraction of coarse aggregates in concrete.

(2) Tortuosity effect. Since coarse aggregates are considered to be non-transport media, chloride ions must detour around the edges of coarse aggregates when migrating through concrete. This leads to a longer migration and diffusion path, reduces the transport velocity of ions, and consequently lowers the chloride diffusion coefficient. When both the dilution effect and the tortuosity effect of coarse aggregates are taken into account, the chloride diffusion coefficient in concrete is given as follows:

(13) $$D_{DT}=D_{m}{\left(1-V_{f}\right)}^{3/2}$$

(3) Interfacial transition zone (ITZ) effect. The interfacial region between the cement mortar and aggregates in concrete is referred to as the interfacial transition zone (ITZ). The ITZ has a composition similar to that of the cement matrix but exhibits different microstructural, morphological, and density characteristics. Owing to the boundary effect and water bleeding effect at the interface between coarse aggregates and cement mortar—which differ in physical and chemical properties—the ITZ possesses high porosity, making it a weak region in terms of both mechanical properties and chloride ingress resistance of cement-based materials. In this study, it is assumed that the chloride diffusion coefficient of the ITZ is the same for all specimens of the same concrete type. When the dilution effect, tortuosity effect, and ITZ effect of coarse aggregates are all taken into account, the chloride diffusion coefficient in concrete is expressed as follows:

(14) $$D=D_{m}{\left(1-V_{f}\right)}^{3/2}+(D_{\mathrm{ITZ}}-D_{m})V_{I}$$

where *D* is the chloride diffusion coefficient accounting for the combined effects of all meso-scale components in concrete; $D_{\mathrm{ITZ}}$ is the chloride diffusion coefficient of the interfacial transition zone (ITZ); and $V_{I}$ is the volume fraction of the ITZ.

The ITZ typically has a very small thickness, generally ranging from 15 to 50 μm [54,55]. However, experimental studies have shown that the chloride diffusion coefficient in the ITZ is often several tens to even hundreds of times higher than that in the cement mortar matrix [56]. At present, the chloride diffusion coefficient of the ITZ in concrete is difficult to measure directly through experimental means and is mostly obtained indirectly through experimental results. It is correlated with the ITZ volume fraction, which is in turn governed by the coarse aggregate content. Accordingly, the ITZ diffusion coefficient can be expressed as a function of the coarse aggregate volume fraction. Wang et al. [57] conducted numerical simulations of concrete constituents from a meso-scale perspective and established the relationships between the ITZ volume fraction and the coarse aggregate volume fraction, as well as between the ITZ diffusion coefficient and the coarse aggregate volume fraction, as follows:

(15) $$V_{I}=14.5849\frac{h_{I}}{s_{M}}V_{f}$$

(16) $$D_{\mathrm{ITZ}}=\left[\frac{1-0.8525V_{f}-{\left(1-V_{f}\right)}^{3/2}}{V_{I}}+1\right]D_{m}$$

where $h_{I}$ is the thickness of the interfacial transition zone (ITZ), taken as 50 μm; and $s_{M}$ is the maximum particle size of coarse aggregates, taken as 25,000 μm in this study.

#### 4.1.3. Effect of the Bond Interface on the Diffusion Coefficient

Analysis of the variation in the bond interface influence coefficient with distance from the bond interface (s) reveals that it exhibits a bell-shaped curve characteristic, with a distribution approximating a Gaussian distribution. Through fitting trials and comparisons, the GaussAmp function is selected as an empirical model to describe the distribution effect of the bond interface influence coefficient. The GaussAmp function, a form of the Gaussian function, is expressed as follows:

(17) $$y=y_{0}+Ae^{-\frac{{\left(x-x_{c}\right)}^{2}}{2w^{2}}}$$

The GaussAmp function produces a symmetric bell-shaped curve centered at its peak. The value increases toward the center and decreases with distance from it. Within a certain range away from the center, the function value drops rapidly. As the independent variable approaches positive infinity or negative infinity, the function value approaches a certain baseline value. In Equation (17), $y_{0}$ is the limit value that the function approaches as the independent variable tends to infinity; *A* is the peak height above $y_{0}$; $x_{c}$ is the center of the curve on the *x*-axis; and w controls the width of the bell-shaped curve and is related to the full width at half maximum.

Based on the characteristics of the function curve and the variation in the bond interface influence coefficient with distance from the bond interface, $y_{0}$ is found to be approximately 1, while $x_{c}$ is taken as 0, corresponding to the bond interface position defined in the coordinate system. Accordingly, the GaussAmp function expression for the bond interface influence coefficient is given as follows:

(18) $$f(i)=1+Ae^{-\frac{s^{2}}{2w^{2}}}$$

**Figure 12:**
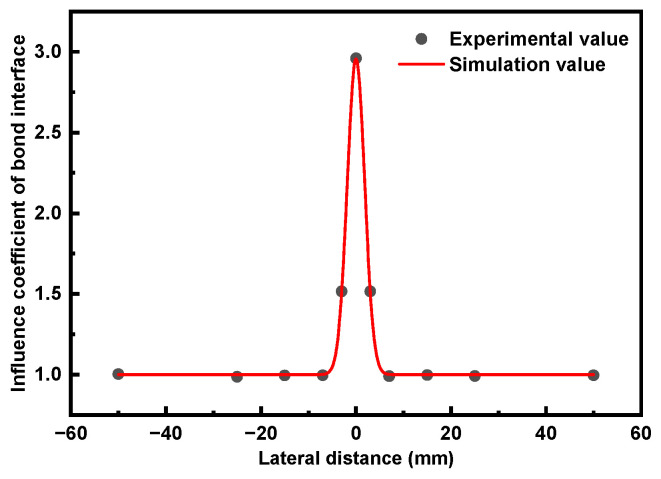
Bond interface influence coefficient at *t* = 30 d.

Fitting calculations were performed on the measured average bond interface influence coefficient for an erosion duration of 30 days, and the results are shown in Figure 12.

It can be observed that the experimental results are in good agreement with the theoretical curve, with a correlation coefficient of $R^{2}=0.9987$. Applying the theoretical expression to the specimen results yields the following functional relationship with the distance from the bond interface s:

(19) $$f(i)=1+1.956e^{-\frac{s^{2}}{6.749}}$$

**Figure 13:**
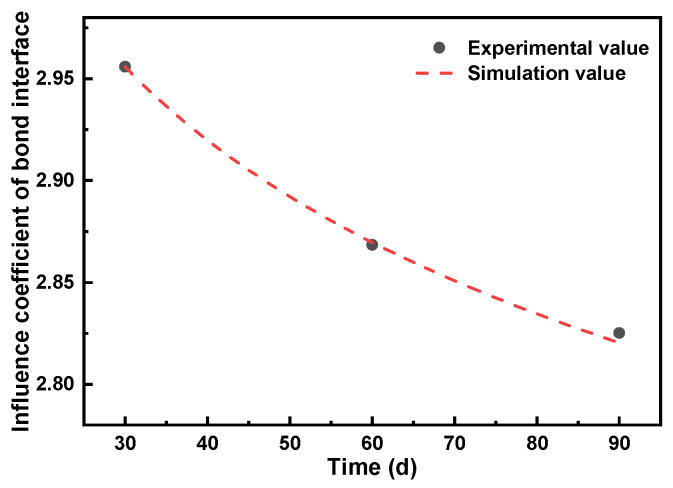
Variation in the bond interface influence coefficient with erosion time.

Considering the time-dependent nature of the bond interface influence coefficient, the average values of the influence coefficient at the bond interface for the three erosion durations were fitted, yielding a correlation coefficient of $R^{2}=0.9997$. The results are presented in Figure 13.

Therefore, the functional relationship between the bond interface influence coefficient and the erosion duration is given as follows:

(20) $$f(i)=f{\left(i\right)}_{ref}\cdot {\left(\frac{t_{ref}}{t}\right)}^{0.0427}$$

where $f{\left(i\right)}_{ref}$ is the bond interface influence coefficient at the reference time $t_{ref}$, and *t* is the erosion duration.

In summary, a comprehensive calculation model for the bond interface influence coefficient at different locations in new and old concrete under different erosion durations is established as follows:

(21) $$f(i)=1+1.956e^{-\frac{s^{2}}{6.749}}\cdot {\left(\frac{30}{t}\right)}^{0.0427}$$

#### 4.1.4. Time-Dependent Effect on the Chloride Diffusion Coefficient

Concrete is a heterogeneous material, and the diffusion rate of chloride ions into concrete is not constant. As erosion time progresses, the hydration process of cementitious materials (e.g., unhydrated cement and reactive admixtures) continues to develop. The newly formed hydration products progressively fill the internal pores of the concrete matrix, thereby gradually reducing the transport pathways for chlorides in concrete. Consequently, the chloride diffusion coefficient decreases with increasing erosion time. Studies [58] have shown that the chloride diffusion coefficient of concrete can be expressed by a power function, as presented in Equation (22):

(22) $$D(t)=D_{ref}\cdot {\left(\frac{t_{ref}}{t}\right)}^{m}$$

where D(t) is the chloride diffusion coefficient at time *t*; $D_{ref}$ is the chloride diffusion coefficient at the reference time $t_{ref}$; and *m* is the time decay factor. The value of *m* depends not only on the properties of the concrete material itself but also on the environmental conditions to which it is exposed. According to the DuraCrete design approach and fib Model Code provisions [50,59,60], recommended values for the diffusion decay exponent are provided. In this study, *m* is taken as 0.37 based on the actual engineering conditions.

By combining Equations (14), (21) and (22), the mathematical model for the chloride diffusion coefficient in new and old concrete is established. This multi-factor model is applicable for evaluating and predicting the chloride diffusion coefficient of new and old concrete with varying volume fractions and maximum coarse aggregate sizes at any given exposure time.

### 4.2. Model Validation

**Figure 14:**
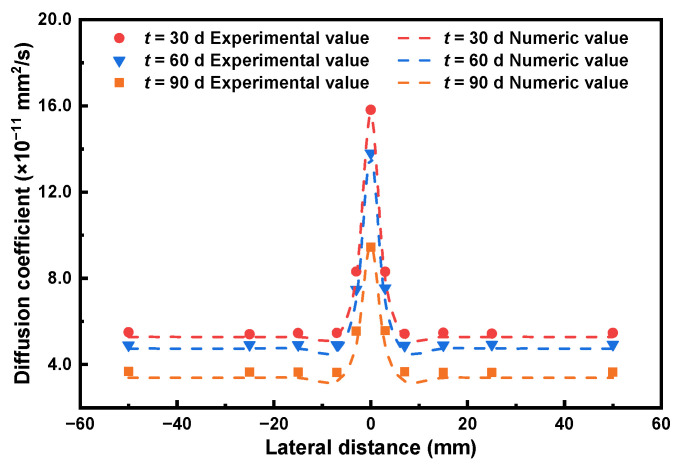
Comparison of chloride diffusion coefficients between numerical model calculations and experimental results.

In this study, the proposed model was used to evaluate the variation in the chloride diffusion coefficient with respect to the distance from the bond interface under different exposure durations for new and old concrete specimens. The predicted results were compared with the experimental data, as shown in Figure 14. Overall, it is observed that the calculated apparent diffusion coefficients from the proposed model show good agreement with the experimental results. The relative error is generally within ±10%, indicating that the developed model for chloride diffusion in new and old concrete is reasonable, accurate, and reliable.

The modeling framework established in this study is general and can be applied to other new-to-old concrete composites and bond interface conditions. However, the fitted bond interface parameters are material-dependent, as they are derived from specific mix proportions, interface treatment procedures, and accelerated laboratory drying–wetting exposure conditions. Recalibration is recommended when the model is extended to different concrete composites, bond interface conditions, or long-term field exposure.

## 5. Conclusions

This study aimed to investigate the influence of the bond interface between new and old concrete on chloride transport under drying–wetting cycle conditions. The chloride concentration distribution in new and old concrete was determined using the potentiometric titration method. Based on the results, a bond interface influence coefficient was proposed to quantify the effect of the bond interface. By incorporating a mesoscale model of concrete, a time-dependent model for the chloride diffusion coefficient in new and old concrete was established. Based on the experimental results and the numerical model proposed in this study, the following main conclusions can be drawn:

(1) Regardless of the erosion duration, the bond interface between new and old concrete consistently exhibits the highest chloride concentration. Chloride ions laterally diffuse from the bond interface into the adjacent concrete on both sides, forming a two-dimensional transport pattern. The chloride concentration exhibits an inverted “V”-shaped distribution near the bond interface. As the distance from the interface increases, the chloride concentration decreases and gradually stabilizes.
(2) At the same depth, the distribution of the bond interface influence coefficient is similar to that of the chloride concentration. As the erosion depth increases, the difference in chloride transport velocity between the bond interface and the adjacent concrete on both sides gradually widens, leading to an increase in the bond interface influence coefficient. As the erosion duration progresses, the concentration difference between the bond interface and the adjacent concrete on both sides decreases, resulting in a decrease in the bond interface influence coefficient.
(3) Based on the GaussAmp fitting of the bond interface influence coefficient curve, a chloride diffusion coefficient model for new and old concrete incorporating the effect of the bond interface was established. The model predictions are in good agreement with the experimental results, validating the accuracy and reliability of the proposed model.

## Figures and Tables

**Table 1:** Chemical composition of cement.

Oxide	SiO_2_	CaO	Al_2_O_3_	Fe_2_O_3_	MgO	K_2_O	Na_2_O	SO_3_	LOI
Content (wt%)	21.6	62.8	1.4	4.6	4.53	0.4	0.5	1.95	2.18

**Table 2:** Physical properties of cement.

Specific Surface Area (m^2^/kg)	Stability	Standard Consistency (%)	Setting Time (min)		Compressive Strength (MPa)	
			Initial Set	Final Set	3 d	28 d
351	Qualified	28	174	221	25.7	50.2

**Table 3:** Physical and chemical properties of fly ash.

Fineness (%)	Density (g/cm^3^)	Water Demand Ratio (%)	Moisture Content (%)	LOI (%)	SO_3_ (%)
9.8	2.2	87	0.2	2.0	2.2

**Table 4:** Physical and chemical properties of GGBS.

Activity Index (%)	Density (g/cm^3^)	Mobility Ratio (%)	Moisture Content (%)	LOI (%)	Chloride Ion Content (%)
82	3.0	97	0.5	2.0	0.05

**Table 5:** Mix proportions of new and old concrete (kg/m^3^) [31].

Concrete Type	Water	Cement	Fine Aggregate	Coarse Aggregate	FA	GGBS	Superplasticizer
Old concrete	176	333	663	1036	71	71	9.5
New Concrete	180	333	690	1035	74	55	8.55

## Data Availability

The original contributions presented in this study are included in the article. Further inquiries can be directed to the corresponding authors.

## References

[1] Anupriya, Bansal P., Graham D.J. (2023). Congestion in Cities: Can Road Capacity Expansions Provide a Solution?. Transp. Res. Part A Policy Pract..

[2] Wang J., Liu C.J., Wu Z.H., Liao R.F., Li G.Z., Lu H.P. (2025). The Roadmap and Strategy for Prioritizing the Development of Public Transport in China. Multimodal Transp..

[3] Zhao P.J. (2010). Sustainable Urban Expansion and Transportation in a Growing Megacity: Consequences of Urban Sprawl for Mobility on the Urban Fringe of Beijing. Habitat Int..

[4] Cui G.Y., Yang Y., Ma C.C., Zhu Z.P., Xiong W., Zhang D.N. (2025). Analysis of scour vulnerability for bridge lifetime based on hydrological uncertainty. Water Resour. Hydropower Eng..

[5] Tu B., Fang Z., Dong Y., Frangopol D.M. (2017). Time-Variant Reliability Analysis of Widened Deteriorating Prestressed Concrete Bridges Considering Shrinkage and Creep. Eng. Struct..

[6] Cao Y., Gehlen C., Angst U., Wang L., Wang Z.D., Yao Y. (2019). Critical Chloride Content in Reinforced Concrete—An Updated Review Considering Chinese Experience. Cem. Concr. Res..

[7] Angst U., Elsener B., Larsen C.K., Vennesland Ø. (2009). Critical Chloride Content in Reinforced Concrete—A Review. Cem. Concr. Res..

[8] Yang S.R., Yang Y.Y., Fang J.J., Mo Z.H., Zhang F., Hu T.G. (2026). Non-stationary evolutionary characteristic analysis of coastal extreme water levels under sea level rise. Water Resour. Hydropower Eng..

[9] Shi W.Z., Najimi M., Shafei B. (2020). Chloride Penetration in Shrinkage-Compensating Cement Concretes. Cem. Concr. Compos..

[10] Park B., Choi Y.C. (2023). Evaluation of Crack Self-Sealing in Flexural Concrete Members with SAPs by Chloride Ion Penetration Resistance. J. Build. Eng..

[11] Geng S., Qiu X., Wu K.R., Deng W.X., Yao Z.D., Li J.P., Yao G.Y., Mei F.P., Zhao J.H. (2026). Research progress on in-fluence of internally incorporated permeable crystalline materials on durability of cement concrete. Water Resour. Hydropower Eng..

[12] Han Q.H., Yang Y.Z., Zhang J.R., Hou D.S., Dong B.Q. (2024). Experimental Investigation and Numerical Simulation of Chloride Diffusion in Rubber Concrete under Dry-Wet Cycles. J. Build. Eng..

[13] Santos D.S., Santos P.M.D., Dias-da-Costa D. (2012). Effect of Surface Preparation and Bonding Agent on the Concrete-to-Concrete Interface Strength. Constr. Build. Mater..

[14] Xia J., Chen K.Y., Hu S.T., Chen J.J., Wu R.J., Jin W.L. (2023). Experimental and Numerical Study on the Microstructure and Chloride Ion Transport Behavior of Concrete-to-Concrete Interface. Constr. Build. Mater..

[15] Huang H.P., Jiang B., Guo T., Fang M.X., Wang T.F., Tu Y.M., Ji Y.H., Wang C., Sas G. (2025). New Insights into the Interfacial Shear Behavior of New-to-Old Concrete: A Molecular Dynamics Simulation Study. Constr. Build. Mater..

[16] Huang H.L., Song X.F., Song X.M., Wu J., Liu H., Chen S.L., Hu J., Wei J.X., Yu Q.J. (2023). A Migrating and Reactive Polycarboxylate Superplasticizer with Coupled Functions of New/Old Concrete Interfacial Agent and Water Reducer. Cem. Concr. Res..

[17] Fu Z.H., Zhu H.B., Guo Z.F., Wen S.Y., Zhang Y. (2025). Splitting Tensile Strength and Microscopic Properties of the Interface between Lightweight Ultra-High Performance Concrete and Normal Concrete: Effects of Old Concrete Strength, Interfacial Roughness, and Lightweight Aggregate Content. Constr. Build. Mater..

[18] Udaipurwala A., Poursaee A., Schiff S.D. (2015). Corrosion Activity in Precast Concrete Elements and Cementitious Closure Pours. J. Bridge Eng..

[19] Li G.P., Hu H., Ren C. (2016). Resistance of Segmental Joints to Chloride Ions. ACI Mater. J..

[20] Li F.M., Luo X.Y. (2019). Interfacial Zone Effects of Chloride Penetration in Precast Concrete Member Joints. Adv. Cem. Res..

[21] Huang G.H., Wu B.Z., Shen Y., Wang L., Li G.P. (2022). Comparative Experiment of Steel Bar Corrosion at Concrete Construction Joints. Front. Mater..

[22] Zhang J.H., Pan Y.T., Li J., Yun H., Guan Z.G. (2024). Experimental Study on Chloride Penetration of the New-to-Old Concrete Interface. Constr. Build. Mater..

[23] Tong L.Y., Šavija B., Zhang M.Z., Xiong Q.X., Liu Q.F. (2025). Chloride Penetration in Concrete under Varying Humidity and Temperature Changes: A Numerical Study. Constr. Build. Mater..

[24] Tong L.Y., Šavija B., Zhang M.Z., Xiong Q.X., Liu Q.F., Hu D. (2024). Study on Time Range of Definite Integral in Derivation Process of Chloride Ion Diffusion Theoretical Model of Concrete. Constr. Build. Mater..

[25] Chen Z., Shi D.D., Cheng X.D. (2025). Fracture characteristics of dredged sand concrete beams in composite salt environment. Water Resour. Hydropower Eng..

[26] Tang L., Nilsson L.-O. (1992). Chloride Diffusivity in High Strength Concrete at Different Ages. Nord. Concr. Res..

[27] Tang L., Nilsson L.-O., Basheer P.A.M. (2011). Resistance of Concrete to Chloride Ingress: Testing and Modelling.

[28] Andrade C., Díez J.M., Alonso C. (1997). Mathematical Modeling of a Concrete Surface “Skin Effect” on Diffusion in Chloride Contaminated Media. Adv. Cem. Based Mater..

[29] Andrade C., Climent M.A., de Vera G. (2015). Procedure for Calculating the Chloride Diffusion Coefficient and Surface Concentration from a Profile Having a Maximum beyond the Concrete Surface. Mater. Struct..

[30] Van der Zanden A.J.J., Taher A., Arends T. (2015). Modelling of Water and Chloride Transport in Concrete during Yearly Wetting/Drying Cycles. Constr. Build. Mater..

[31] Wang K., Wang B.J., Guo J.J., Meng Q.X., Feng H. (2025). Sharpey’s Fiber-Inspired Gradient Anchoring and Multiscale Synergistic Modification of the New–Old Concrete Interface for Bridge Widening Applications. Dev. Built Environ..

[32] (2011). Specification for Mix Proportion Design of Ordinary Concrete.

[33] Wang K., Guo J.J., Yang L. (2021). Effect of Dry–Wet Ratio on Sulfate Transport-Reaction Mechanism in Concrete. Constr. Build. Mater..

[34] Cao J.R., Jin Z.Q., Ding Q.J., Xiong C.S., Zhang G.Z. (2022). Influence of the Dry/Wet Ratio on the Chloride Convection Zone of Concrete in a Marine Environment. Constr. Build. Mater..

[35] Wang K., Guo J.J., Yang L., Zhang P., Xu H.Y. (2022). Multiphysical Damage Characteristics of Concrete Exposed to External Sulfate Attack: Elucidating Effect of Drying–Wetting Cycles. Constr. Build. Mater..

[36] Chen C.H., Chen Y.C., He J., Zhu P.H., Liu R.G., Wang X.J. (2024). Chloride Ion-Induced Deterioration in Concrete under Dry-Wet Cycling Using the Air-Drying Method. Constr. Build. Mater..

[37] Gao Y.H., Zhang J.Z., Zhang S., Zhang Y.R. (2017). Probability Distribution of Convection Zone Depth of Chloride in Concrete in a Marine Tidal Environment. Constr. Build. Mater..

[38] Zhang Y., Wu S.Y., Zhang Y.R., Zhou C.S., Fu C.Q. (2023). Similarities and Probability Distributions of Chloride Convection Zone Depth in Concrete Exposed to Cyclic Drying-Wetting Environments. Cem. Concr. Compos..

[39] Bao J.W., Wei J.N., Zhang P., Zhuang Z.J., Zhao T.J. (2022). Experimental and Theoretical Investigation of Chloride Ingress into Concrete Exposed to Real Marine Environment. Cem. Concr. Compos..

[40] (2013). Technical Specification for Test of Chloride Ion Content in Concrete.

[41] (2019). Standard for Design of Concrete Structure Durability.

[42] (2019). Technical Standard for Inspection of Building Structure.

[43] Beushausen H., Höhlig B., Talotti M. (2017). The Influence of Substrate Moisture Preparation on Bond Strength of Concrete Overlays and the Microstructure of the OTZ. Cem. Concr. Res..

[44] Fu Z.H., Zhu H.B., Guo Z.F., Wen S.Y., Chen Z.H. (2024). Shear Strength of the Interface between Full Lightweight Ceramsite Concrete and Normal Concrete: Effect of Interfacial Roughness and Pouring Time Interval. Constr. Build. Mater..

[45] Sun Y.-M., Liang M.-T., Chang T.-P. (2012). Time/Depth Dependent Diffusion and Chemical Reaction Model of Chloride Transportation in Concrete. Appl. Math. Model..

[46] Babaee M., Castel A. (2018). Chloride Diffusivity, Chloride Threshold, and Corrosion Initiation in Reinforced Alkali-Activated Mortars: Role of Calcium, Alkali, and Silicate Content. Cem. Concr. Res..

[47] Zhang J.Z., Wu H.Y., Du S.S., Li X.X., Zhang Y.R., Wu L.J. (2024). Similarity Analysis of Randomness in Instantaneous Chloride Diffusion Coefficient of Concrete under Different Environmental Exposures. Case Stud. Constr. Mater..

[48] Tian Z.S., Ji H.D., Tian Y., Ye H.L. (2025). Quantifying Anisotropic Chloride Diffusion Coefficients of Interfacial Transition Zone in Concrete. Cem. Concr. Compos..

[49] Meira G.R., Andrade C., Alonso C., Borba J.C., Padilha M. (2010). Durability of Concrete Structures in Marine Atmosphere Zones—The Use of Chloride Deposition Rate on the Wet Candle as an Environmental Indicator. Cem. Concr. Compos..

[50] FIB (2013). Model Code for Concrete Structures 2010.

[51] Garboczi E.J., Bentz D.P. (1997). Analytical Formulas for Interfacial Transition Zone Properties. Adv. Cem. Based Mater..

[52] Sun G.W., Zhang Y.S., Sun W., Liu Z.Y., Wang C.H. (2011). Multi-Scale Prediction of the Effective Chloride Diffusion Coefficient of Concrete. Constr. Build. Mater..

[53] Yang C.C., Cho S.W. (2003). Influence of Aggregate Content on the Migration Coefficient of Concrete Materials Using Electrochemical Method. Mater. Chem. Phys..

[54] Li C.Z., Song X.B. (2022). Mesoscale Modeling of Chloride Transport in Unsaturated Concrete Based on Voronoi Tessellation. Cem. Concr. Res..

[55] Nežerka V., Bílý P., Hrbek V., Fládr J. (2019). Impact of Silica Fume, Fly Ash, and Metakaolin on the Thickness and Strength of the ITZ in Concrete. Cem. Concr. Compos..

[56] Wu K., Han H., Li H.X., Dong B.Q., Liu T.J., De Schutter G. (2021). Experimental Study on Concurrent Factors Influencing the ITZ Effect on Mass Transport in Concrete. Cem. Concr. Compos..

[57] Wang Y.Z., Wu L.J., Wang Y.C., Liu C.X., Li Q.M. (2018). Effects of Coarse Aggregates on Chloride Diffusion Coefficients of Concrete and Interfacial Transition Zone under Experimental Drying-Wetting Cycles. Constr. Build. Mater..

[58] Thomas M.D.A., Bamforth P.B. (1999). Modelling Chloride Diffusion in Concrete: Effect of Fly Ash and Slag. Cem. Concr. Res..

[59] DuraCrete (2000). General Guideline for Durability Design and Redesign.

[60] Gjørv O. (2011). Durability of Concrete Structures. Arab. J. Sci. Eng. AJSE.

